# Strain Heterogeneity and Extended Defects in Halide Perovskite Devices

**DOI:** 10.1021/acsenergylett.4c00921

**Published:** 2024-05-29

**Authors:** Kieran W. P. Orr, Jiecheng Diao, Krishanu Dey, Madsar Hameed, Miloš Dubajić, Hayley L. Gilbert, Thomas A. Selby, Szymon J. Zelewski, Yutong Han, Melissa R. Fitzsimmons, Bart Roose, Peng Li, Jiadong Fan, Huaidong Jiang, Joe Briscoe, Ian K. Robinson, Samuel D. Stranks

**Affiliations:** ∇ Department of Physics, Cavendish Laboratory, University of Cambridge, JJ Thomson Avenue, Cambridge CB3 0HE, U.K.; × Department of Chemical Engineering and Biotechnology, University of Cambridge, Philippa Fawcett Drive, Cambridge CB3 0AS, U.K.; § Center for Transformative Science, ShanghaiTech University, Shanghai 201210, China; ∥ School of Engineering and Materials Science, Queen Mary University of London, Mile End Road, London E1 4NS, U.K.; ⊥ Diamond Light Source, Harwell Science and Innovation Campus, Fermi Avenue, Didcot OX11 0DE, U.K.; # London Centre for Nanotechnology, University College London, London WC1E 6BT, U.K.; ⊗ Condensed Matter Physics and Materials Science Department, Brookhaven National Laboratory, Upton, New York 11793, United States

## Abstract

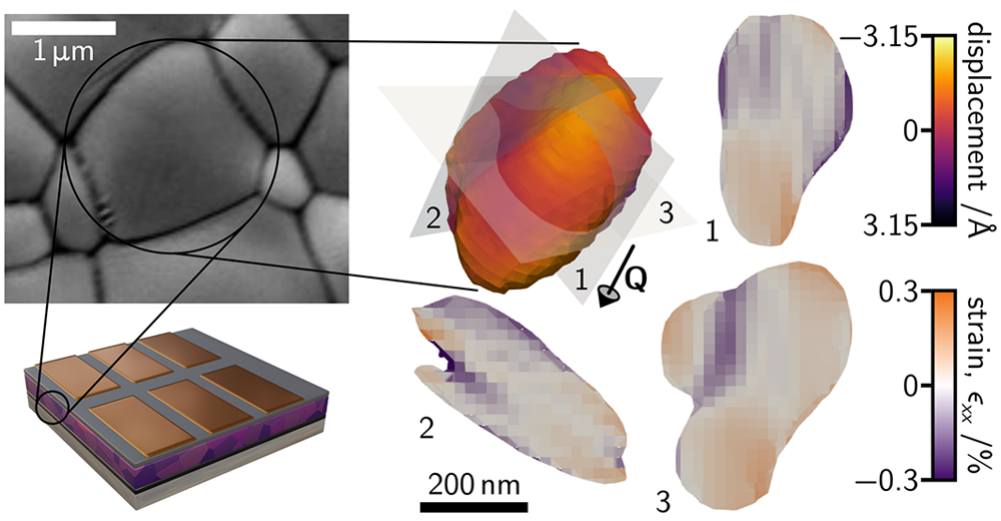

Strain is an important property in halide perovskite semiconductors used for optoelectronic applications because of its ability to influence device efficiency and stability. However, descriptions of strain in these materials are generally limited to bulk averages of bare films, which miss important property-determining heterogeneities that occur on the nanoscale and at interfaces in multilayer device stacks. Here, we present three-dimensional nanoscale strain mapping using Bragg coherent diffraction imaging of individual grains in Cs_0.1_FA_0.9_Pb(I_0.95_Br_0.05_)_3_ and Cs_0.15_FA_0.85_SnI_3_ (FA = formamidinium) halide perovskite absorbers buried in full solar cell devices. We discover large local strains and striking intragrain and grain-to-grain strain heterogeneity, identifying distinct islands of tensile and compressive strain inside grains. Additionally, we directly image dislocations with surprising regularity in Cs_0.15_FA_0.85_SnI_3_ grains and find evidence for dislocation-induced antiphase boundary formation. Our results shine a rare light on the nanoscale strains in these materials in their technologically relevant device setting.

The prodigious rise in efficiencies of optoelectronic devices based on halide perovskites over the last two decades^1,2^ has attracted significant scientific attention. In addition, the comparative ease with which halide perovskite materials and devices can be synthesized (using spin-coating techniques, for example) has produced a diverse and energetic community focused on improving device performance still further. With so many researchers refining fabrication procedures, often in an empirical manner, it is of paramount importance to understand how each synthetic step affects the optoelectronic properties of the light-absorbing halide perovskite material. For example, bulk tensile strain in the in-plane directions of halide perovskite films increases with annealing temperature due to a difference in thermal expansion coefficient between glass substrates and the comparatively soft halide perovskite.^3^ This is important, because strain is known to modulate bandgap,^4^ carrier dynamics,^5^ material stability,^6^ and device longevity,^7^ among other properties.^8^ However, such a film-average description of strain is inadequate given the structural and performance heterogeneities across multiple length scales (nm–cm) in halide perovskite films.^9−11^ Adjacent device layers can also stress the perovskite layer, generating additional local strains.^12^

Further, the vast majority of halide perovskites used in emerging solar cell technologies are formed as polycrystalline thin films—a morphology they share with numerous other functional materials used in catalysis,^13,14^ LEDs,^15^ optical coatings,^16^ and batteries.^17^ The strain tolerance of such materials intimately depends not only on long-range heterogeneities and grain boundaries, but also on the intragrain material structure.^18,19^ As such, understanding the grain and subgrain strain states in polycrystalline halide perovskite materials is critical for achieving mechanically robust devices with long-term operational stabilities.

Bragg coherent diffraction imaging (BCDI) is a (typically synchrotron-based) X-ray diffraction technique that can be used to image atomic displacement vectors, **u**(**r**), within a diffracting object from which local strains can be calculated.^20^ Using iterative phase retrieval algorithms to overcome the well-known phase problem of X-ray diffraction, “reconstructions” of a diffracting object’s electron density can be obtained from coherent diffraction patterns. In general, these reconstructions are complex, containing both real and imaginary components, with their modulus proportional to the object’s electron density and their phase (or argument) proportional to the size of the atomic displacements along the scattering vector direction, **Q**, in each voxel. A difference in phase of 2π between two points corresponds to a difference in displacement of the relevant lattice spacing, . The relevant average, “unstrained” values for *d* are calculated from Rietveld refinements of powder X-ray diffraction data shown in Figure S1 of the Supporting Information (SI). BCDI has been used to study strain distributions in ZnO nanorods,^21^ (dis)solution of calcite,^22^ and to monitor dislocation dynamics in LiNiMn_1.5_O_4_ battery electrodes.^23^ Ferroelastic domain structures have also been identified in CsPbBr_3_ halide perovskite nanoparticles with BCDI,^24^ and our recent study employed the technique to track the increased dislocation migration in MAPbBr_3_ microcrystals in response to visible light illumination.^25^

Here, by performing BCDI measurements on Cs_0.1_FA_0.9_Pb(I_0.95_Br_0.05_)_3_ and Cs_0.15_FA_0.85_SnI_3_ (FA = CH(NH_2_)_2_) halide perovskites in full device stacks, we find large (up to *ca*. 1%) intragrain strains with root-mean-squared local strain values of up to *ca*. 0.5%. We also show that strain is not only highly heterogeneous within grains, but also from grain to grain. By interrogating the local strain distributions in Cs_0.15_FA_0.85_SnI_3_, we uncover a surprisingly high incidence of dislocations. The nanoscale (BCDI spatial resolution = *ca*. 10 nm) structural insights presented here are uncommon in the halide perovskite field, especially for films sandwiched between other functional layers in a full device architecture.

Cs_0.1_FA_0.9_Pb(I_0.95_Br_0.05_)_3_ is a typical high-performance mixed-component Pb-based halide perovskite formulation used in solar cells, photodetectors, and LEDs and is broadly representative of the widely employed FAPbI_3_-rich family of materials. In this work, we use a n-i-p device architecture of ITO/SnO_2_/Cs_0.1_FA_0.9_Pb(I_0.95_Br_0.05_)_3_/Spiro-MeOTAD/Au. The Cs_0.1_FA_0.9_Pb(I_0.95_Br_0.05_)_3_ films investigated here were post-treated with aerosolised DMF which causes grain growth, a reduction in defect concentrations, and better stability compared to untreated references.^26−28^ We also explore Sn-based perovskites in an ITO/PEDOT:PSS/Cs_0.15_FA_0.85_SnI_3_/C_60_/BCP/Cu p-i-n solar cell device architecture, where this perovskite composition shows promise in low bandgap cells^29^ (*e.g*. for use in all-perovskite tandems), in LEDs,^30^ and are also employed in field-effect transistors.^31,32^ Devices based on these compositions were chosen to provide a broad overview of the likely structural chemistry at play across different halide perovskites within the broad material family noting, for example, that the chemistries of Pb- and Sn-based systems are often different. Aerosol treatment of Pb-based films and the specific 100% Sn composition were chosen for the resulting large morphological grain sizes which ensure the high counts at the detector required for successful BCDI reconstruction and, in general, higher radiation tolerance as the grain surface/volume ratio is smaller. Sample quality is confirmed with powder X-ray diffraction patterns, photoluminescence spectra, scanning electron microscopy (SEM) morphological data, and JV characteristics as presented in Figures S1–S4 of the SI.

Figure 1 summarizes the approach we devised for this work. A schematic of the measurement setup is presented in Figure 1a where coherent X-rays are incident through the top metal contacts and transport layer to illuminate halide perovskite grains below. The contacts are thinner than standard devices to minimize X-ray absorption, with 60 nm thickness for the Au contacts on Cs_0.1_FA_0.9_Pb(I_0.95_Br_0.05_)_3_-based devices, and 25 nm for the Cu contacts on Cs_0.15_FA_0.85_SnI_3_-based devices. The diffracted signal then leaves the device through those same top layers on the way to the detector. For each grain studied, many coherent diffraction patterns are collected at finely spaced incidence angles along a rocking curve. One such pattern is shown in the inset of Figure 1a. Figure 1b shows a schematic of the device architectures with the individual layers for the Cs_0.1_FA_0.9_Pb(I_0.95_Br_0.05_)_3_- and Cs_0.15_FA_0.85_SnI_3_-based devices specified. An example SEM image of the Cs_0.1_FA_0.9_Pb(I_0.95_Br_0.05_)_3_ perovskite is given in Figure 1c showing morphological grain sizes of *ca*. 0.5–2 μm. Finally, Figure 1d is a reconstruction of an example Cs_0.1_FA_0.9_Pb(I_0.95_Br_0.05_)_3_ grain colored according to the size of the atomic displacements along the direction of the scattering vector, **Q**, shown.

**Figure 1:**
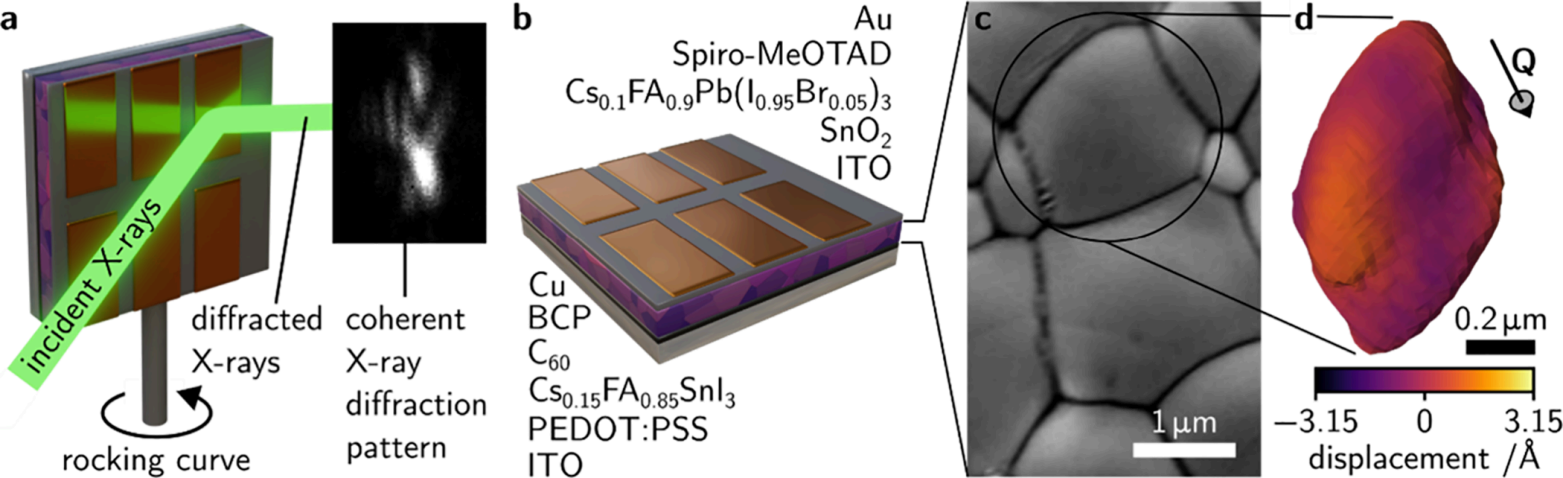
**Nanoscale strain characterization in full perovskite solar cell devices with BCDI.** (**a**) Schematic of the BCDI experiment for imaging strain in individual halide perovskite grains in full device stacks with an example coherent diffraction pattern shown as an inset. (**b**) Solar cell device architecture schematic. Layers comprising the Pb-based (top) and Sn-based (bottom) devices considered in this work are given. (**c**) Scanning electron micrograph of Cs_0.1_FA_0.9_Pb(I_0.95_Br_0.05_)_3_ film morphology showing the large grain sizes. (**d**) Reconstruction of an example grain of Cs_0.1_FA_0.9_Pb(I_0.95_Br_0.05_)_3_, colored according to the size of the atomic displacements along the scattering vector direction, **Q**. This figure is illustrative of our experimental procedure; we note that the reconstruction shown in panel **d** does not correspond to the specific grain highlighted in **c**. ITO ≡ indium tin oxide. Sprio-MeOTAD ≡ 2,2′,7,7′-tetrakis(N,N-di-*p*-methoxyphenylamine)-9,9′-spirobifluorene. BCP ≡ bathocuproine. PEDOT:PSS ≡ Poly(3,4-ethylenedioxythiophene):poly(styrenesulfonate).

In our coordinate system, **Q** is approximately coincident with the *x* Cartesian direction, and according to the tensorial description of strain, taking the spatial derivative of the atomic displacements with respect to the scattering vector direction (*x* direction) yields a local value for one of the diagonal elements of the strain tensor, ϵ_*xx*_.^33^ This corresponds to local values of tensile/compressive (positive/negative) strain in our crystal between voxels along the scattering vector direction. An example of such a treatment is given in Figure 2a for an example Cs_0.1_FA_0.9_Pb(I_0.95_Br_0.05_)_3_ grain where the top left image is of the grain’s reconstructed electron density in 3D, colored according to the size of the atomic displacement, as before. Once the atomic displacement field is differentiated with respect to the *x* (≡**Q**) direction, we may take slices through the grain’s volume to visualize the grain’s internal strain distribution. Such slices are also presented in Figure 2a with their position in the grain’s reconstruction indicated by the numbered gray planes. We see that the intragrain strain distribution is highly heterogeneous with distinct islands of tensile/compressive strain of *ca*. 100 nm in size. Note that many other Cs_0.1_FA_0.9_Pb(I_0.95_Br_0.05_)_3_ grains are also analyzed in this work and are presented in Figure S5 of the SI.

**Figure 2:**
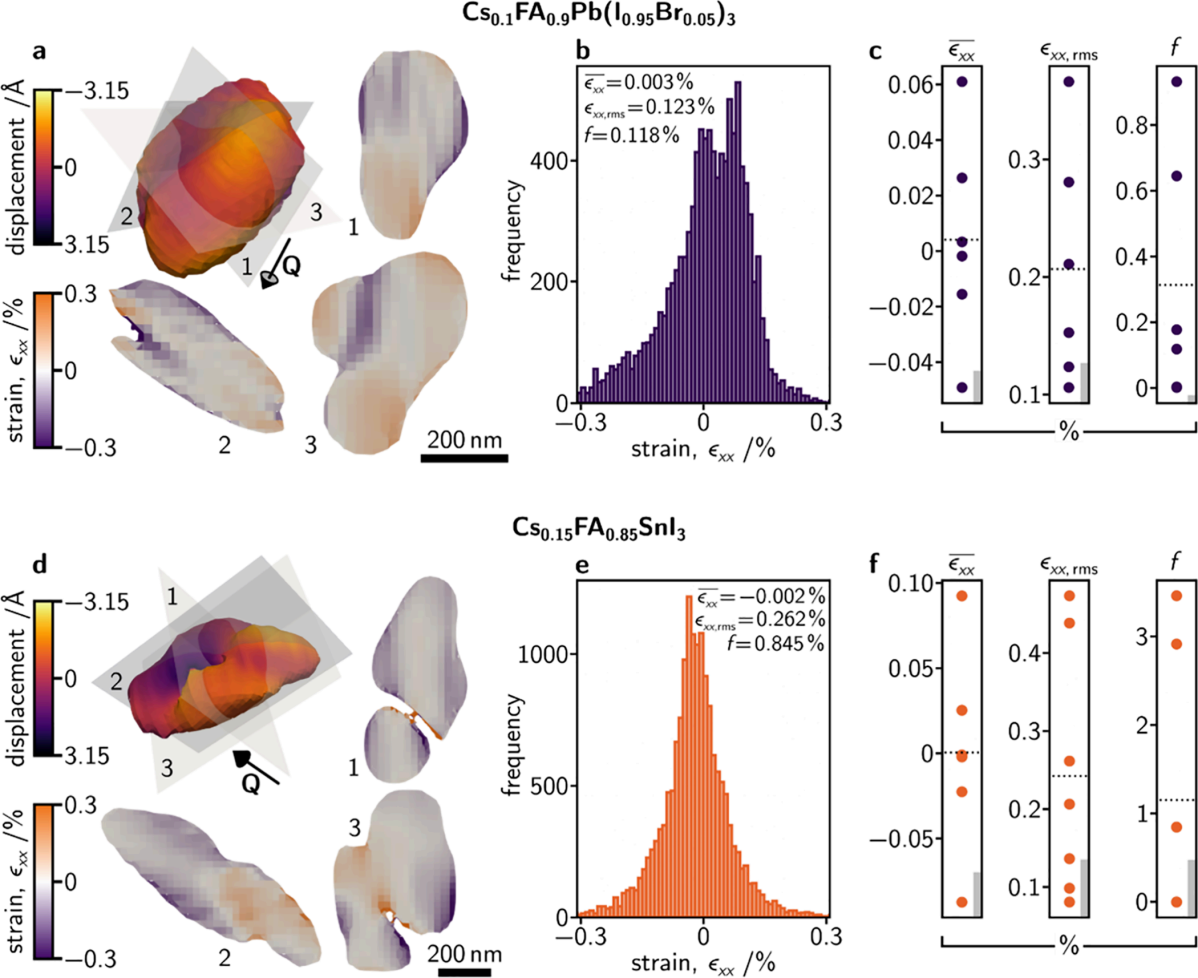
**Halide perovskite grains contain large and heterogeneous strains.** (**a**) 3D electron density reconstruction of a Cs_0.1_FA_0.9_Pb(I_0.95_Br_0.05_)_3_ grain colored according to the size of atomic displacements along the scattering vector direction, **Q**, and three numbered orthogonal slices colored according to their local tensile/compressive strains (also along the **Q** direction). (**b**) A histogram of all local strains (voxel by voxel) of the reconstruction shown in **a**. (**c**) Statistical descriptors (mean strain, ϵ̅_*xx*_; root mean squared strain, ϵ_*xx*,rms_; and fraction of the crystal, ***f***, where |ϵ_*xx*_| > 1%) of the strain distribution within all the Cs_0.1_FA_0.9_Pb(I_0.95_Br_0.05_)_3_ grains measured. All values are in %, the dashed line shows the mean, and the gray bars in the bottom left of the plots give an estimate of the uncertainty due to beam-induced changes (see Figure S7 and Table S1, SI). (**d**, **e**, **f**) The same as for panels **a**, **b**, **c** but for Cs_0.15_FA_0.85_SnI_3_ grains. The additional reconstructions leading to data in panels **c** and **f** are presented in Figures S5 and S6 of the SI.

To quantitatively describe the strain distribution in each grain, we plot histograms of the local strain values, ϵ_*xx*_, and calculate the mean, ϵ̅_*xx*_, root-mean-square, ϵ_*xx*,rms_, and fraction, *f*, (in percent) of the crystal volume that is more strained than 1%. This cutoff value for *f* is chosen to highlight that, for some grains, there are small but significant portions of the crystals with strains above this threshold and because such strains of >1% would significantly impede the performance of more well-established semiconductors such as Si^34^ and Cu(In,Ga)Se_2_.^35^ A histogram of local tensile/compressive strains, ϵ_*xx*_, in the reconstructions shown in Figure 2a is given in Figure 2b. ϵ̅_*xx*_, ϵ_*xx*,rms_, and *f* for all the Cs_0.1_FA_0.9_Pb(I_0.95_Br_0.05_)_3_ grains measured are plotted in Figure 2c. For Cs_0.1_FA_0.9_Pb(I_0.95_Br_0.05_)_3_; *ca*. −0.05% < ϵ̅_*xx*_ < *ca*. 0.06%, *ca*. 0.1% < ϵ_*xx*,rms_ < *ca*. 0.4%, and 0 < *f* < *ca*. 0.9%. The same analysis is applied to the Cs_0.15_FA_0.85_SnI_3_ grains where Figure 2d shows the three-dimensional reconstruction of an example grain, and slices through it highlighting the heterogeneous internal strain distribution. Figure 2e shows the histogram of local strains for this grain, with its ϵ̅_*xx*_, ϵ_*xx*,rms_, and *f* given, and Figure 2f plots ϵ̅_*xx*_, ϵ_*xx*,rms_, and *f* for every Cs_0.15_FA_0.85_SnI_3_ grain studied (see Figure S6, SI). For Cs_0.15_FA_0.85_SnI_3_, *ca*. −0.09% < ϵ̅_*xx*_ < *ca*. 0.10%, *ca*. 0.05% < ϵ_*xx*,rms_ < *ca*. 0.45%, and 0 < *f* < *ca*. 3.5%. Not only are the values for ϵ̅_*xx*_, ϵ_*xx*,rms_, and *f* plotted in Figure 2c and f relatively high, they also vary significantly from grain to grain, indicating that (i) the intragrain strain distribution is distinctly heterogeneous, and (ii) that strain varies significantly on a grain-to-grain basis (Figures S5 and S6, SI).

The gray bars in the bottom right corners of the plots in Figure 2c and f are estimates of the uncertainty arising from any X-ray beam-induced effects which are considered in greater detail in the SI (Figure S7 and Table S1). While beam damage could not be totally excluded from our measurements, the calculated uncertainties are small with respect to both the absolute values and spread of the strain distribution descriptors calculated, therefore we do not attribute the variability we observe to the result of beam damage.

The intragrain strain distributions for both systems considered are similar, but with Cs_0.15_FA_0.85_SnI_3_ showing overall higher strain and strain heterogeneities. Additionally, in the slices through the Cs_0.15_FA_0.85_SnI_3_ grain shown in Figure 2d, we observe some voids. Such voids in reconstructions are generally indicative of defects and they have low reconstructed electron density because they do not have the same crystal structure as the rest of the grain and hence do not diffract X-rays to the detector. By rendering the reconstructions for three Cs_0.15_FA_0.85_SnI_3_ grains partially transparent, we can see the (curvi)linear shapes of these defects which are highlighted with black lines in Figure 3a–c. The reconstruction shown in Figure 3a is the same as that in Figure 2d but viewed from a different angle. Such one-dimensional crystal defects are confirmed as dislocations from the phase (atomic displacement) discontinuity in their local displacement fields.^36^

**Figure 3:**
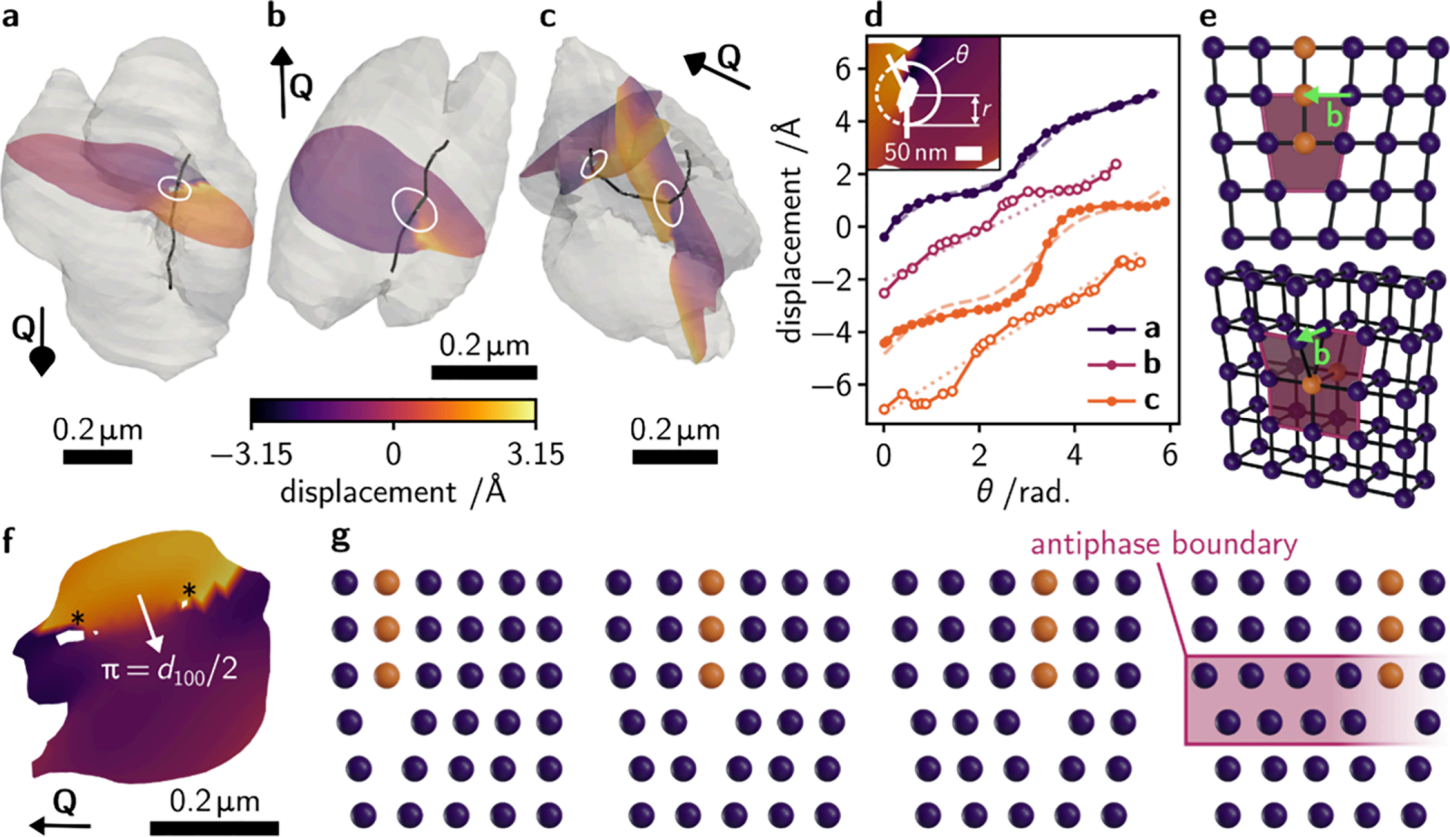
**Extended defects in Cs**_**0.15**_**FA**_**0.85**_**SnI**_**3**_**halide perovskite grains.** (**a**, **b**, **c**) Electron density reconstructions for three grains of Cs_0.15_FA_0.85_SnI_3_ with dislocations shown in black. Slices through the reconstructions colored according to atomic displacements are highlighted. The scattering vector directions, **Q**, scale bars, and white circles indicating the path for displacement vs. arc angle data extraction are shown. (**d**) Circles and solid lines: displacement vs. arc angle, *θ*, data for each of the dislocations shown in panels **a**, **b**, and **c**. Filled circles: data taken from a plane containing **Q** for edge dislocation characterization, empty circles: data taken from a plane perpendicular to **Q** for screw dislocation characterization. Dashed lines: fits to the data of the function given in Equation S3 for an edge dislocation. Dotted lines: fit to the data of the function given in Equation S4 for a screw dislocation (see SI for Equations). Data are vertically offset for clarity. **Inset:** Illustration of the displacement vs. arc angle data extraction. (**e**) Schematics of edge (top) and screw (bottom) dislocations with Burgers vectors, **b**, shown. (**f**) Another slice through the grain shown in panel **c** showing the presence of an antiphase boundary from the π phase jump. Asterisks indicate the reconstruction voids at the center of the grain’s dislocation. (**g**) Schematic of a possible antiphase boundary formation mechanism from edge dislocation glide. The spheres that form the extra atomic plane of the edge dislocation are colored orange. Different columns of atoms form the dislocation as the glide proceeds. Spheres would correspond to the corner-sharing lead halide octahedra in the perovskite crystal structure.

We can quantitatively describe the dislocations by taking slices through the reconstructions and then noting the values of atomic displacement as a function of arc angle, θ, as we travel around the dislocation core at a given radius*r*. Example slices are shown colored according to the size of the atomic displacements in Figure 3a–c, with the method for the extraction of local strain information is shown diagrammatically in the inset of Figure 3d. Resulting displacement vs. arc angle plots for the three dislocations are presented in Figure 3d. Depending on the type of dislocation—edge or screw, depicted schematically in Figure 3e—the local strain field will be different. Details of the quantitative dislocation analysis and of the functions fit to the displacement vs. arc angle data are given in the SI but, in summary, the plots shown in Figure 3d will show a sinusoidal modulation (filled circles) for a pure edge dislocation and will be linear for a pure screw dislocation (open circles).

As illustrated in Figure 3e, the Burgers vector is perpendicular to the dislocation line for an edge dislocation but is parallel to it for a screw dislocation. A dislocation may be of mixed character if the angle between the Burgers vector and the dislocation line is between these two extremes. The resulting displacement vs. arc angle plot for a mixed dislocation will, accordingly, possess neither a perfect sinusoidal modulation nor a constant gradient. When dislocations bend, as is obvious in Figure 3c, portions of the dislocation are, therefore, necessarily mixed in character, i.e. they comprise both screw and edge character. For the three dislocations identified in Figure 3a–c, the magnitude of the phase discontinuity is *ca*. 2π such that the Burgers vector magnitudes are, respectively, 6.52 Å, 5.70 Å, and 7.08 Å (edge) and 7.27 Å (screw) (Table S2, SI), all of which are reasonably close to the *d*_100_ pseudocubic lattice spacing of 6.29 Å refined from powder X-ray diffraction data (Figure S1, SI). Therefore, each of these dislocations is likely to be a ⟨100⟩ dislocation with the one in Figure 3a having predominantly edge character, the one in Figure 3b having predominantly screw character, and the one in Figure 3c having both edge and screw character depending on the portion of the dislocation considered. We do note, however, that full, unambiguous dislocation characterization requires multipeak BCDI where the sizes of atomic displacements are known in three dimensions,^37−40^ not just along the scattering vector for the single Bragg peak measured as in our experiment.

We find ⟨100⟩ dislocations in three out of the seven Cs_0.15_FA_0.85_SnI_3_ grains studied, suggesting that such dislocations are a common structural feature in Sn-based halide perovskite devices (we do not find any such dislocations in the Cs_0.1_FA_0.9_Pb(I_0.95_Br_0.05_)_3_ grains studied). ⟨100⟩ and ⟨110⟩ dislocations have been identified in MAPbBr_3_,^25^ and ⟨100⟩ and ⟨110⟩ dislocations have been imaged in FAPbI_3_,^41^ but this is the first time such extended defects have been uncovered in a full device stack. This is a unique advantage of the X-ray BCDI technique over, for example, destructive etch pit methods^42,43^ or electron microscopy-based methods^41^ which would be unable to penetrate through the adjacent device layers without significant difficulty.

Additionally, Figure 3f shows a further slice taken through the reconstruction shown in Figure 3c. The dislocation appears to bound a region which a *ca*. π phase offset from the rest of the crystal. This phase offset corresponds to a relative atomic displacement of  between these two regions,^37,44^ which is indicative of a {100} antiphase boundary, the structure of which is highlighted in the right-hand schematic of Figure 3g. The remainder of Figure 3g schematically illustrates a possible antiphase boundary formation mechanism akin to the one uncovered by Ahmed et al.^45^ stemming from the glide of a ⟨100⟩ dislocation such as the one also present in this grain.

We have previously connected higher dislocation densities and increased strain heterogeneities in MAPbBr_3_ microcrystals with beam-induced material degradation and modified luminescence properties (blue-shift in photoluminescence spectrum and longer photoluminescence lifetimes).^25^ Similarly, by tuning dislocation densities in epitaxially grown CsPbBr_3_ samples, Jiang et al. found dislocation assisted recombination to be as important as grain boundary or point defect assisted recombination in the material’s transient photoluminescence properties.^46^ In addition, increased microstrain, which is the manifestation of the strain heterogeneities we have probed in bulk average X-ray diffraction, has been correlated with reduced film stability and device performance in alloyed compositions based on FAPbI_3_.^47,48^ These results indicate that local strain heterogeneity and extended defects markedly affect the performance and stability of halide perovskite semiconductors, with this work newly observing these dislocations and nanoscale strain fields in full solar cell device architectures with polycrystalline thin film absorbers. The stark difference in the numbers of dislocations in the Pb- and Sn-based systems studied suggests composition is important for determining dislocation densities, with their prevalence one possible reason why Sn-based halide perovskites remain problematic. Future device work will be needed to minimize the presence of dislocations through optimization of composition, architecture, and processing conditions.

The interplay between grain boundaries and dislocations is critical for understanding and controlling the mechanical stability of materials. Such considerations are particularly pertinent to the fracture resistance of halide perovskites given their proposed use in flexible devices.^49−51^ For example, it has been proposed that the densities of Frank-Read dislocation sources and dislocation accumulation at film surfaces^52^ can modify fracture toughness.^53^ Performing single-grain BCDI measurements on gold polycrystalline films, Yau et al. imaged increased grain growth in the area local to a dislocation during annealing.^54^ These effects are important because of the ability of grain size to affect material strength under Hall-Petch theory where smaller grains generally yield stronger materials.^55,56^ It has been suggested that halide perovskites may be brittle because dislocations cannot migrate across grain boundaries efficiently, hindering plastic deformation and dislocation management has been identified as a promising avenue for intrinsic material toughening.^57^ A heterogeneous strain distribution within grains will also cause heterogeneities in the propensities of dislocations to migrate due to the different elastic energies of a dislocation’s local strain field superposed on the grain’s underlying strain distribution. Hence, further investigation of the implications of the subgrain heterogeneities reported here on dislocation migration (which is increased when solar cells are operated under illumination^25^) will produce further interesting insights that can be applied to improve device longevity.

Practically for device makers, the mechanical properties of materials can be tuned during thermal recrystallization processes.^58^ Adapted annealing processes have been shown to remove threading dislocations in inorganic semiconductors, for example annealing Ge under forming gas (a mixture of H_2_ and N_2_),^59^ and post-processing photolithography patterning and annealing of ZnSe on GaAs.^60^ However, we note that elevated annealing temperatures cause detrimental in-plane tensile strains in halide perovskites due to the aforementioned thermal expansion mismatch between perovskite and substrate.^3^ As such, solution-based recrystallization, already demonstrated on halide perovskites,^61,62^ but with no explicit consideration of dislocations, should be given greater attention. In this work, since no dislocations are found in Cs_0.1_FA_0.9_Pb(I_0.95_Br_0.05_)_3_ grains, it is possible that the aerosol post-treatment used on these samples has a defect-healing effect that removes dislocations. Though further studies are required to confirm this hypothesis, contact potential difference, current/voltage, and photoluminescence measurements suggest such aerosol post-treatments reduce defect densities and prolong film performance.^26,28,63^ The observation that dislocation formation is a key feature of beam damage in MAPbBr_3_^25^ is consistent with the supposition that dislocation annihilation through improved processing enhances halide perovskite performance.

For the samples considered here,^26,31^ and generally for halide perovskite used in devices, the grain sizes are sufficiently large and films sufficiently thin to be only one grain thick. This fact, combined with the reflection geometry of the experiment, means the adjacent transport layers will touch the grains at either end of the scattering vector, **Q**. However, we do not see any systematic strain fields emanating from the transport layer/perovskite interfaces and, in any case, the possible twin images resulting from each reconstruction process makes us unable to distinguish which transport layer is at either end of the scattering vector. It is feasible that the precise strain-inducing effect of adjacent layers changes with processing conditions, indeed this is the principle on which interface engineering^64^ is predicated, and future work will be directed toward understanding such effects.

To put the size of the strains observed here further into context, for strained epitaxy, lattice mismatches greater than *ca*. 2% give critical thicknesses (beyond which further growth is incommensurate) of only up to *ca*. 10 nm, yielding layers that are prohibitively thin for most applications (including the optoelectronic ones that concern halide perovskites).^65^ i.e. beyond *ca*. 2% strained materials are unlikely to form coherent crystals larger than nanoparticles. Additionally, given the ability of strain to influence the band structure in halide perovskites,^4,8^ the local strain heterogeneities uncovered here mean that charge carriers are likely to experience a varying electronic environment as they diffuse through the perovskite. Therefore, strain is very likely to be at least partly responsible for the heterogeneous carrier dynamics^66^ and charge carrier extraction observed at the interface between halide perovskite films and transport layers in devices.^18^ Experiments such as ours that can characterize strain heterogeneities in three dimensions in full devices, are thus a key tool for assessing how different fabrication procedures of both the halide perovskite absorber and adjacent layers can help to homogenize strain and carrier extraction to yield higher performances. Such studies, using this technique as a platform for strain characterization, will be the subject of future work. Furthermore, our demonstration of BCDI on full device stacks indicate the feasibility of full *operando* studies on solar cells investigating the effects of illumination and bias on plastic deformation-facilitating dislocation migration in perovskite thin films, though care must be taken to ensure the effects of beam damage are minimized.

We note that this approach is unable to resolve whether the tensile/compressive strain observed is due to purely mechanical effects, or to compositional inhomogeneity (i.e., chemical strain), or both in such mixed-component systems. For example, local lattice expansion could be the result of a greater concentration of larger FA ions over smaller Cs ions, or the result of external stresses. BCDI only measures the atomic displacement field, and so correlative experiments with elemental mapping techniques using X-ray fluorescence (XRF), electron energy dispersive spectroscopy (EDS), or electron energy loss spectroscopy (EELS), should be a future research focus. Nonetheless, pronounced strain heterogeneities have also been identified within single crystals^25^ and individual grains^19^ of nonalloyed halide perovskites, and so the results presented here are unlikely to be solely due to compositional heterogeneity. We are also blind to any overall uniform tensile/compressive strain across the entire grain, which would be determined by Bragg peak shifts relative to a reference “unstrained” film. Though, such an “unstrained” reference can be nebulous to define given that, for example, thermal annealing needs to take place to produce thin films appropriate for comparison. All diffraction peaks measured for this work appear on the same powder ring with the detector in the same position, indicating that there is negligible change in uniform underlying tensile or compressive strain between grains in our samples.

In conclusion, by carrying out BCDI measurements on full halide perovskite device stacks, we have uncovered strikingly heterogeneous strain distributions within individual grains at the nanoscale and that the local strain distribution changes markedly from grain to grain. While the Cs_0.15_FA_0.85_SnI_3_ grains are overall more strained and more heterogeneous than their Cs_0.1_FA_0.9_Pb(I_0.95_Br_0.05_)_3_ counterparts, the size of the intragrain strains between the two systems is similar, suggesting our results are broadly applicable to a wide array of solution-processed halide perovskite thin films. By interrogating the local strain fields in the immediate vicinity of defect sites, we also discover a notable difference between the Cs_0.1_FA_0.9_Pb(I_0.95_Br_0.05_)_3_ and Cs_0.15_FA_0.85_SnI_3_ systems, in that dislocation defects are remarkably prevalent in the latter tin-based halide perovskite which will likely affect film performance and stability. Exploring halide perovskite composition space in greater detail will be the subject of future studies. Our results offer unique insight into the nanoscale material properties of halide perovskite films buried in full solar cell devices unavailable from measurements of average structure from conventional X-ray diffraction. Such information is important because subgrain, grain-to-grain, and nanoscale structures will need to be targeted to fully understand the remarkable optoelectronic properties of halide perovskites.^67^

## Experimental Methods

### Fabrication of Cs_0.1_FA_0.9_Pb(I_0.95_Br_0.05_)_3_-Based Devices

SnO_2_: The SnO_2_ colloid precursor was obtained from Alfa Aesar (tin(IV) oxide, 15% in H_2_O colloidal dispersion). The solution was diluted in H_2_O to 2.5% (5 mL water into 1 mL 15% tin oxide solution). The final solution was spin coated onto ITO substrates at 3000 rpm for 30 s, and then heated on a hot plate in ambient air at 150 °C for 30 min.

#### Perovskite Solutions and Deposition

The precursor solution of the double-cation (CsFA) perovskite with nominal chemical composition Cs_0.1_FA_0.9_Pb(I_0.95_Br_0.05_)_3_ was prepared by co-dissolving cesium iodide (CsI, 0.13 M, Dyesol), lead(II) iodide (PbI_2_, 1.105 M), lead bromide (PbBr_2_, 0.195 M, TCI) and formamidinium iodide (FAI, 1.17 M) in a mixed solvent of DMF and *N*-Methyl-2-pyrrolidone (NMP) (4:1 in volume ratio). The solution was stirred at 60 °C for 1 h and was passed through a 0.45 μm PTFE filter before use. 60 μL of precursor solution was dropped on the SnO_2_-coated ITO substrate and spun at 4000 rpm for 20 s. 0.6 mL of antisolvent was dropped at the 10th second.

#### Aerosol Treatment

All films were preannealed at 100 °C for 2 min to dry most of the solvent prior to aerosol treatment. Films were then placed within the preheated reactor, with the temperature set at 100 °C. The treatment was carried out by flowing aerosolised DMF into the reactor at 0.3 dm^3^ min^–1^ for 5 min. The aerosol was generated using a piezoelectric generator. For two standard devices the substrates were placed in the central section of the reactor, approximately 4 cm from the aerosol inlet. After the 5 min had elapsed, the aerosol flow was switched to N_2_ and the samples were left on the heated graphite block for a further 5 min at the same temperature to remove the remaining DMF in the chamber. Samples were then left to cool and were removed and placed into a glovebox for additional thermal annealing for 60 min.

#### Fabrication of Remaining Layers

SpiroMeOTAD: The Spiro-MeOTAD organic layer was spin-coated on the perovskite film at 3000 rpm for 30 s. The Spiro-MeOTAD solution was synthesized by dissolving 72.3 mg of Spiro-MeOTAD in 1 mL anhydrous chlorobenzene with additives of 56 μL of *tert*-butylpyridine (tBP) and 34 μL lithium bis (trifluoromethylsulfonyl) imide salt (520 mg/mL) in acetonitrile.

Gold contacts: 60 nm-thick gold electrodes were added by using thermal evaporation under vacuum. Evaporation rates were: 0.1 Ås^–1^ for the first 3 nm, 0.4 Ås^–1^ for the next 7 nm, and 1 Ås^–1^ thereafter.

### Fabrication of Cs_0.15_FA_0.85_SnI_3_-Based Devices

Cs_0.15_FA_0.85_SnI_3_-based devices were fabricated according to the following procedure adapted from Senanayak, Dey, and co-workers.^31,68^

#### Materials

All the starting perovskite precursors, *viz*. formamidinium iodide (FAI, GreatCell Solar Materials), cesium iodide (CsI, Sigma-Aldrich, anhydrous, beads, −10 mesh, 99.999% trace metals basis), tin iodide (SnI_2_, Sigma-Aldrich, anhydrous, beads, −10 mesh, 99.99% trace metals basis), and tin fluoride (SnF_2_, Sigma-Aldrich) were used without any further purification. Poly(3,4-ethylenedioxythiophene):poly(styrenesulfonate) (PEDOT:PSS) aqueous solution (Clevois P VP AI 4083) was purchased from Heraeus Co. Ltd. C_60_ and bathocuproine (BCP) were purchased from Creaphys GmbH and Ossila, respectively.

#### Fabrication

Prepatterned Glass/ITO substrates (10–15 Ω cm^–2^, Kintec) were cleaned with a 15 min ultrasonic bath in detergent (decon 90), water, acetone and isopropanol, followed by drying with a nitrogen gun. After UV ozone treatment of 15 min, the PEDOT:PSS hole transport layer (HTL) was fabricated from an aqueous dispersion which was filtered through a 0.45 μm PVDF filter and then spin coated at 4000 rpm for 30 s. The films were then annealed at 120 °C for 20 min and taken immediately into a glovebox (H_2_O < 0.1 ppm, O_2_ < 0.1 ppm) for subsequent deposition of perovskite films. For Cs_0.15_FA_0.85_SnI_3_ perovskite films, all the relevant precursors (FAI, CsI, SnI_2_, and SnF_2_) were dissolved in appropriate ratios in a mixed solvent of DMF and DMSO (3:1 by volume) to form a 1.5 M solution and then left for stirring at room temperature for 2–3 h. Following this, perovskite solutions were filtered through 0.22 μm PTFE syringe filters and then spin-coated on PEDOT-coated ITO substrates at 5000 rpm (acceleration: 7000 rpms^–1^) for 25 s, with chlorobenzene (antisolvent) dropped on the spinning substrates 10 s before the end. The as-coated perovskite films were then annealed at 100 °C for 10 min. It is noted that a constant amount of SnF_2_ (10 mol % with respect to the amount of Sn in the solution) was added in the solution to suppress the formation of Sn vacancies. Subsequently, 20 nm of C_60_, 7 nm of BCP, and 25 nm of Cu were sequentially deposited by thermal evaporation inside a glovebox to complete the p-i-n devices.

### Thin Film Synthesis

Bare Pb-based and Sn-based thin films were prepared following the procedures outlined above, but spin-coating only the perovskite thin film directly onto glass substrates coated with ITO.

### BCDI Measurements

BCDI measurements were carried out at the I13-1 beamline of the Diamond Light Source (U.K.) using an X-ray beam energy of 11.8 keV (beam flux at sample: *ca*. 1 × 10^7^ photons s^–1^). Diffraction patterns were collected using the beamline’s Excalibur photon-counting direct X-ray detector with a pixel size of 55 μm (Medipix3 chip) which was at a distance of 2.83 m from the sample. Measurements were taken in reflection geometry. The beam was focused with a Fresnel zone plate and the sample was placed after the focal plane to give a spot of *ca*. 2.5 μm in diameter.

In a typical measurement, coherent diffraction patterns around the 100 Bragg peak were collected at 51 rocking curve angles separated by 0.005 ° (spanning a total angle range of 0.25 °) with a collection dwell time of 10 s at each rocking curve angle. Measurements were conducted at room temperature.

### Electron Density Reconstruction

The measured coherent diffraction patterns were fast Fourier transformed back to real space to get the crystal reconstructions. The amplitude was restored by taking the square-root of the intensity, while the phase was retrieved using iterative phasing methods. A 300 iteration loop linear combination of typical iterative phasing algorithms were used, including error reduction (ER), hybrid input-output (HIO),^69^ and relaxed averaged alternating reflection (RAAR)^70^ algorithms. The shrink-wrap^71^ method was applied for updating the real space constraints during the final 100 iterations and guided algorithms^72^ and 10 parallel reconstructions with random seeds were run, selecting the solution with maximum sharpness after each of the 10 generations. Each diffraction pattern was reconstructed ten times with random initial guess to ensure reproducibility.

Reconstructions shown in this work are isovolumes whose surface is determined by setting a threshold value of electron density modulus and not displaying regions of space with modulus lower than this threshold value. For most reconstructions, a threshold of 0.1 was used (reconstructed electron density functions are normalized between 0 and 1).

### Reconstruction Analysis

Electron density reconstructions were produced in .vtk file format and viewed and analyzed using the open-source Paraview data visualization software.^73^

## Supporting Information Available

Additional data: powder X-ray diffraction, photoluminescence spectroscopy, scanning electron microscopy, current/voltage characterization, additional BCDI grain reconstructions, local strain distributions, local strain histograms, discussion of X-ray beam damage, theory of Burgers vector determination for edge and screw dislocations, and supplementary experimental methods (PDF)
