# Supplementary material for: Strain Heterogeneity and Extended Defects in Halide Perovskite Devices
Source: ACS Energy Lett. 2024 May 29;9(6):3001–11. doi: 10.1021/acsenergylett.4c00921 (PMC11190982; doi:10.1021/acsenergylett.4c00921)
Supplement: Supplementary file 1 — nz4c00921_si_001.pdf [file nz4c00921_si_001.pdf]

Strain Heterogeneity and Extended Defects in Halide Perovskite Devices

SUPPORTING INFORMATION

Kieran W. P. Orr[1,2], Jiecheng Diao[3], Krishanu Dey†[1], Madsar Hameed[4], Miloš Dubajić[2], Hayley L. Gilbert[2,5], Thomas A. Selby[2], Szymon J. Zelewski‡[1,2], Yutong Han[2], Melissa R. Fitzsimmons[2], Bart Roose [2], Peng Li[5], Jiadong Fan[3], Huaidong Jiang[3], Joe Briscoe[4], Ian K. Robinson[6,7], Samuel D. Stranks*[1,2]

[1] *Department of Physics, Cavendish Laboratory, University of Cambridge, JJ Thomson Avenue, Cambridge, CB3 0HE, U.K.*
[2] *Department of Chemical Engineering and Biotechnology, University of Cambridge, Philippa Fawcett Drive, Cambridge, CB3 0AS, U.K.*
[3] *Center for Transformative Science, ShanghaiTech University, Shanghai 201210, China*
[4] *School of Engineering and Materials Science, Queen Mary University of London, Mile End Road, London E1 4NS, U.K.*
[5] *Diamond Light Source, Harwell Science and Innovation Campus, Fermi Ave, Didcot OX11 0DE, U.K.*
[6] *London Centre for Nanotechnology, University College London, London WC1E 6BT, U.K.*
[7] *Condensed Matter Physics and Materials Science Department, Brookhaven National Lab, Upton, New York 11793, United States*

* sds65@cam.ac.uk

† Current address: *Department of Physics, Clarendon Laboratory, University of Oxford, Oxford, OX1 3PU, U.K.*
‡ Current address: *Department of Experimental Physics, Faculty of Fundamental Problems of Technology, Wroclaw University of Science and Technology, 50-370, Wroclaw, Poland*

Contents:

Confirmation of sample quality 2
BCDI reconstructions of grains not shown in the main text 4
Discussion of beam damage 7
Determination of Burgers vector magnitudes 8
Additional experimental methods 10
References 11

Confirmation of sample quality:

Presented below are standard characterisations (powder X-ray diffraction, photoluminescence spectroscopy, scanning electron microscopy, and device current/voltage measurements) to confirm the quality of the samples used in this study.

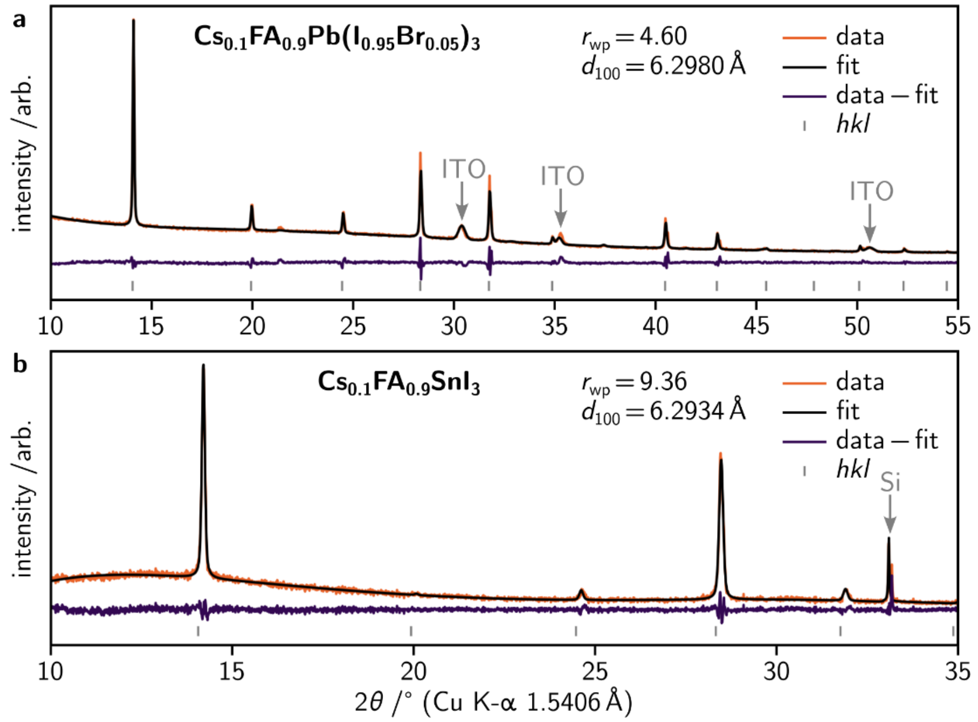

Fig. S1: Pawley fits of powder X-ray diffraction measurements of **a** $Cs_{0.1}FA_{0.9}Pb(I_{0.95}Br_{0.05})_3$ and **b** $Cs_{0.15}FA_{0.85}SnI_3$ films prepared on ITO-coated glass and Si respectively. (Pseudo-cubic) d_{100} lattice parameters and r_{wp} fit parameters are quoted and grey *hkl* marks indicate the positions of pseudo-cubic perovskite Bragg peaks. Refinements were performed using $Pm\overline{3}m$ symmetry using Topas Academic software (v7).[1]

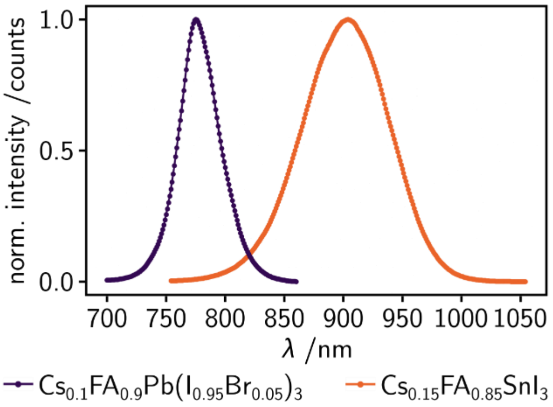

Fig. S2: Photoluminescence emission spectra (405nm excitation) of $Cs_{0.1}FA_{0.9}Pb(I_{0.95}Br_{0.05})_3$ (purple) and $Cs_{0.15}FA_{0.85}SnI_3$ (orange) films. Spectra are concordant with those given in other literature.[2,3] Spectra have been normalised after subtraction of a flat background.

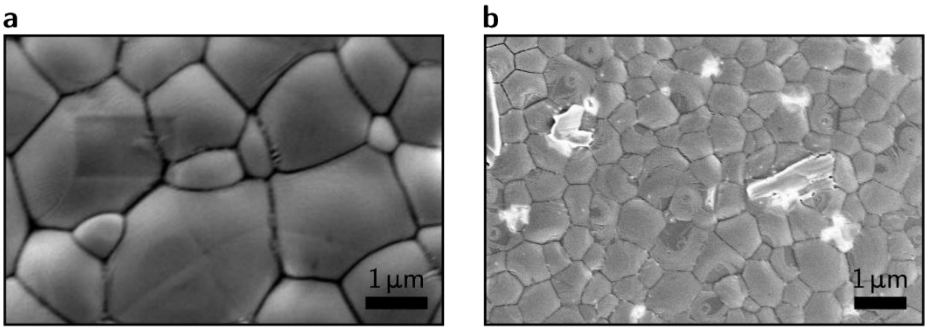

Fig. S3: Scanning electron micrographs of representative **a** $Cs_{0.1}FA_{0.9}Pb(I_{0.95}Br_{0.05})_3$ and **b** $Cs_{0.15}FA_{0.85}SnI_3$ films. In both cases, morphological grain sizes are on the order of 0.5–2.0µm. This is slightly larger than average for halide perovskite devices but chosen to better suit the BCDI technique as mentioned in the main text. A cropped region of panel **a** is used in Fig. 1c of the main text.

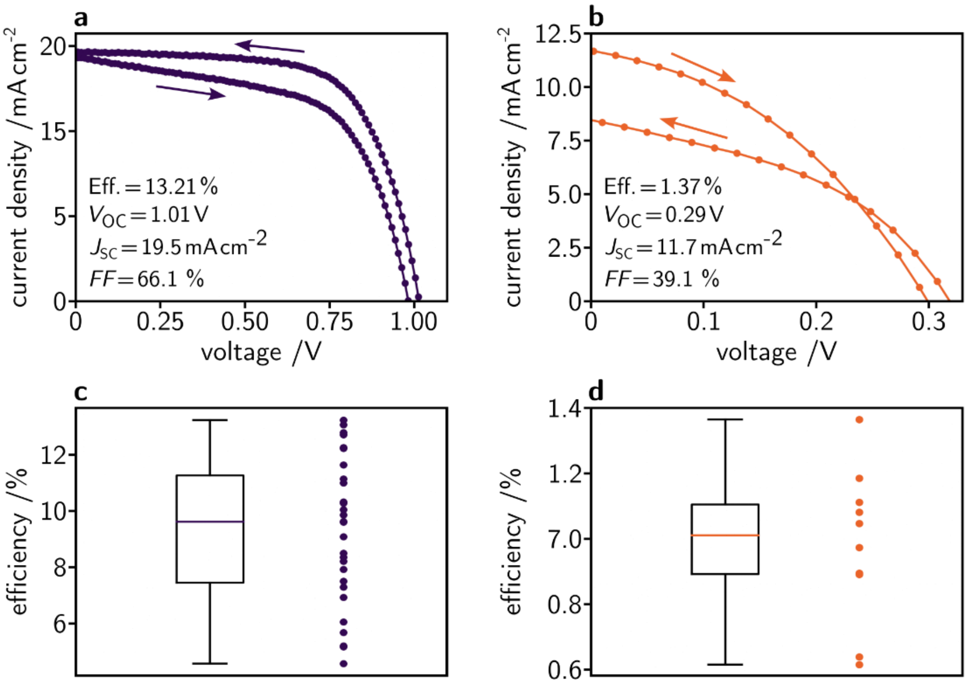

Fig. S4: JV characteristics for champion **a** $Cs_{0.1}FA_{0.9}Pb(I_{0.95}Br_{0.05})_3$-based and **b** $Cs_{0.15}FA_{0.85}SnI_3$-based solar cells from batches representative of the devices considered in this work. Box and whisker plots of these **c** $Cs_{0.1}FA_{0.9}Pb(I_{0.95}Br_{0.05})_3$-based and **d** $Cs_{0.15}FA_{0.85}SnI_3$-based solar cell batches with individual efficiencies for each cell plotted separately on the right. $Cs_{0.1}FA_{0.9}Pb(I_{0.95}Br_{0.05})_3$ devices for JV characterisation have 100nm-thick Au contacts (as opposed to the 60nm-thick contacts used for the BCDI measurements) to reflect what is more commonly used in the field. Similarly, $Cs_{0.15}FA_{0.85}SnI_3$ devices for JV characterisation have 120nm-thick Cu contacts (as opposed to the 25nm-thick contacts used for the BCDI measurements). Thinner contacts were used on the beamline to as not to attenuate the X-ray beam any more than necessary.

BCDI reconstructions of grains not shown in the main text:

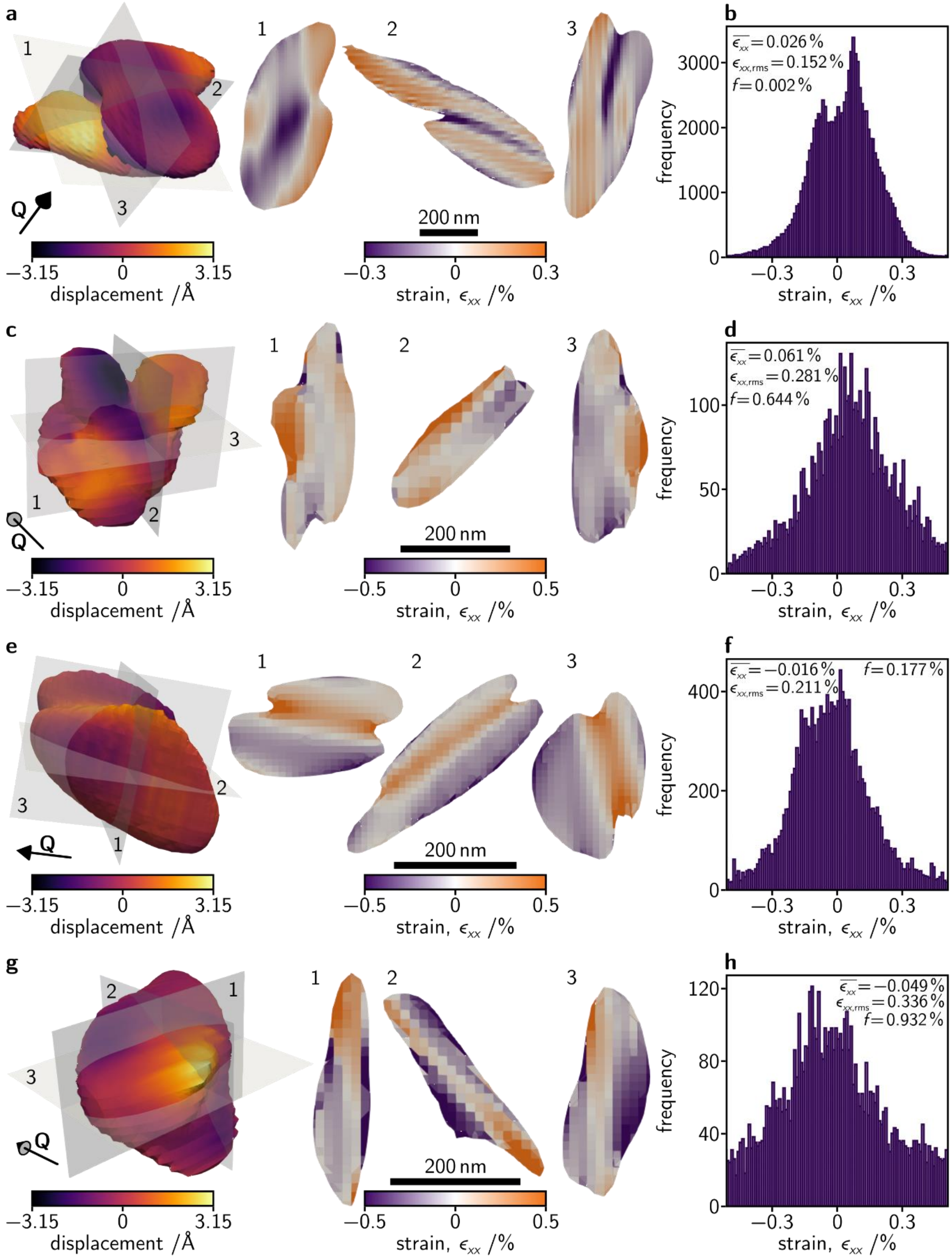

Fig. S5: **a**, **c**, **e**, **g** Reconstructions of individual $Cs_{0.1}FA_{0.9}Pb(I_{0.95}Br_{0.05})_3$ grains and corresponding slices showing their internal strain distributions not shown in the main text. **b**, **d**, **f**, **h** Histograms of local strain for the grains shown in the same row. $\overline{\epsilon_{xx}}$, $\epsilon_{xx,\mathrm{rms}}$, and f strain distribution descriptors are quoted.

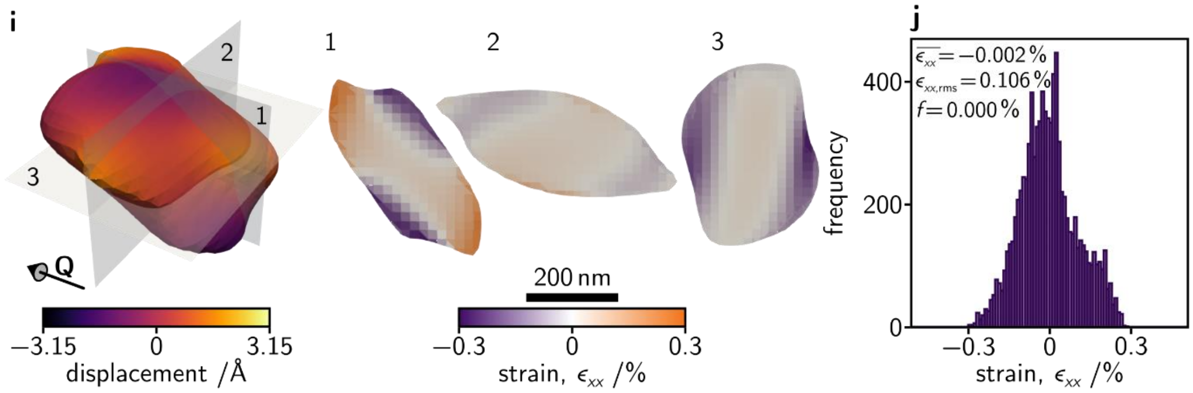

Fig. S5 continued: **i** Reconstruction of an individual $Cs_{0.1}FA_{0.9}Pb(I_{0.95}Br_{0.05})_3$ grain and corresponding slices showing its internal strain distributions not shown in the main text. **j** Histogram of local strain for the grain shown in the same row. $\overline{\epsilon_{xx}}$, $\epsilon_{xx,\mathrm{rms}}$, and f strain distribution descriptors are quoted.

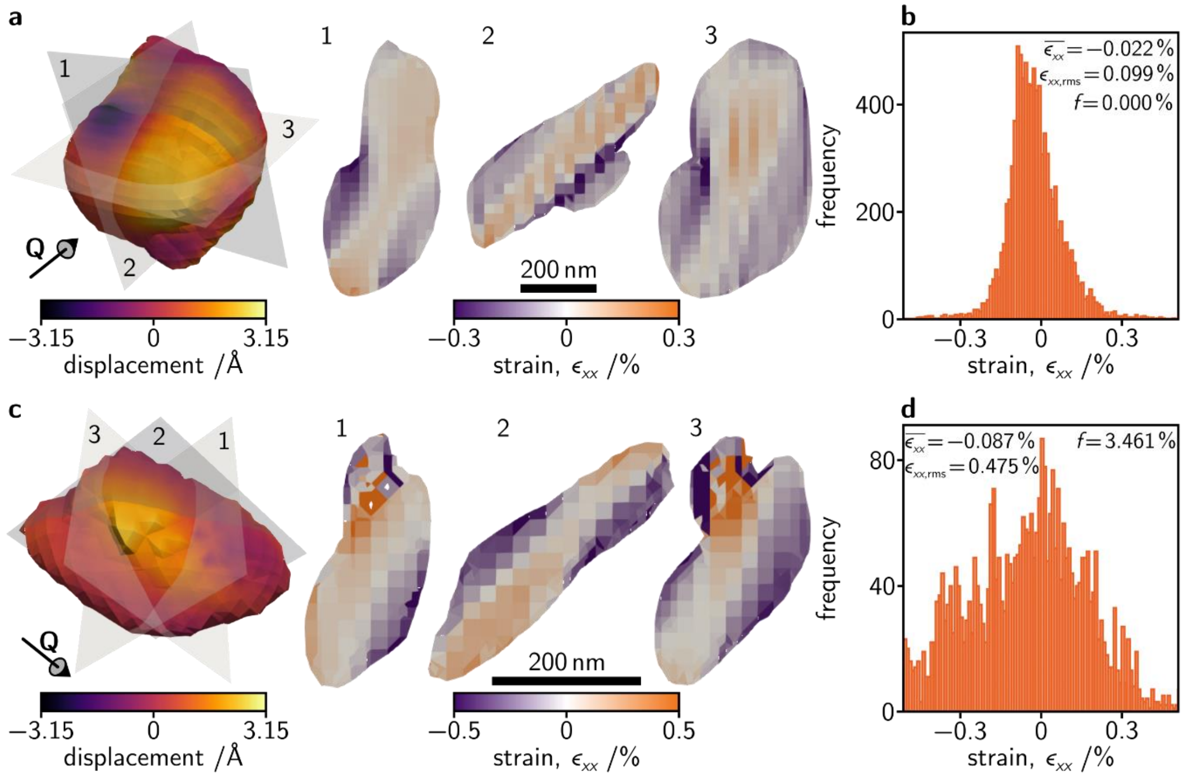

Fig. S6: **a**, **c**, Reconstructions of individual $Cs_{0.15}FA_{0.85}SnI_3$ grains and corresponding slices showing their internal strain distributions not shown in the main text. **b**, **d**, Histograms of local strain for the grains shown in the same row. $\overline{\epsilon_{xx}}$, $\epsilon_{xx,\mathrm{rms}}$, and f strain distribution descriptors are quoted.

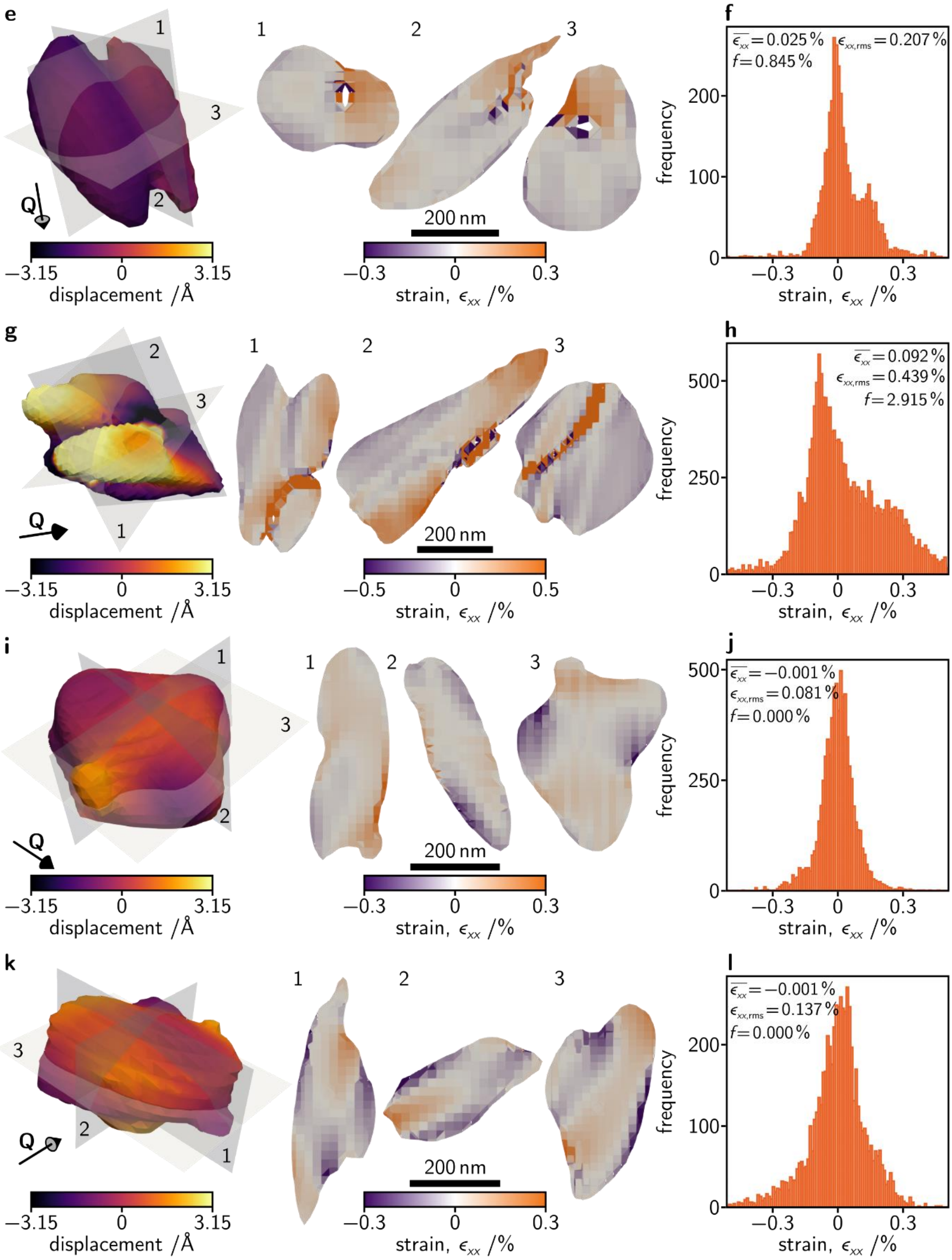

Fig. S6 continued: **e**, **g**, **i**, **k** Reconstructions of individual $Cs_{0.15}FA_{0.85}SnI_3$ grains and corresponding slices showing their internal strain distributions not shown in the main text. **f**, **h**, **j**, **l**, Histograms of local strain for the grains shown in the same row. $\overline{\epsilon_{xx}}$, $\epsilon_{xx,\mathrm{rms}}$, and f strain distribution descriptors are quoted.

Discussion of beam damage:

It is well known that halide perovskites are X-ray-sensitive materials. As such, here we explicitly consider the effect of X-ray-induced changes on the grains studied. The beam energy used of 11.8keV has been empirically determined to minimise beam damage during previous beamline visits.

For all the grains where we took multiple BCDI measurements, we have calculated the local strain distribution descriptors and plotted them as a function of scan number in Fig. S7. Following this, we have calculated the mean magnitude of the changes from successive measurements to get an estimate of the likely size of the effects of beam damage for each of the values plotted in Fig. 2c and Fig. 2f of the main text. These means are tabulated in Table S1 along with the standard error of the mean magnitudes of beam-induced changes. We consider the mean absolute change as an "error bar due to beam-induced changes", and it is plotted as a grey bar in the bottom right corner of the plots in Fig. 2c & 2f of the main text. We have considered the $Cs_{0.1}FA_{0.9}Pb(I_{0.95}Br_{0.05})_3$ and $Cs_{0.15}FA_{0.85}SnI_3$ grains separately, as it appears that the $Cs_{0.1}FA_{0.9}Pb(I_{0.95}Br_{0.05})_3$ grains undergo smaller beam-induced changes.

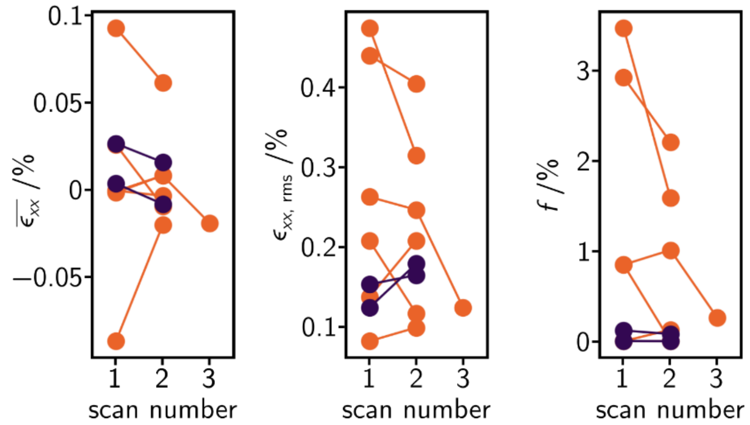

Fig. S7: Local strain distribution descriptors as a function of BCDI scan number for grains where multiple measurements were taken. Values for $Cs_{0.1}FA_{0.9}Pb(I_{0.95}Br_{0.05})_3$ are shown in purple and those for $Cs_{0.15}FA_{0.85}SnI_3$ are shown in orange.

$Cs_{0.1}FA_{0.9}Pb(I_{0.95}Br_{0.05})_3$

Local strain distribution descriptor	$\overline{\epsilon_{xx}}$ /%	$\epsilon_{xx,\mathrm{rms}}$ /%	f /%
Mean change magnitude	0.011	0.033	0.020
Standard error in the mean change magnitude	0.000(4)	0.015	0.013

$Cs_{0.15}FA_{0.85}SnI_3$

Local strain distribution descriptor	$\overline{\epsilon_{xx}}$ /%	$\epsilon_{xx,\mathrm{rms}}$ /%	f /%
Mean change magnitude	0.026	0.073	0.639
Standard error in the mean change magnitude	0.007	0.019	0.226

Table S1: Mean change magnitudes and standard error in the mean change magnitudes for local strain distribution descriptors upon successive exposure to X-rays.

Determination of Burgers vector magnitudes:

When fitting the displacement *vs.* arc angle data, the raw data should be collected at a radius, r, from the dislocation core small enough such that the strain field due to the dislocation dominates over any other longer-range strain fields present in the crystal, but also large enough to sample enough points around the dislocation core. Practically, in this work a radius of 50nm was chosen as standard, though 35nm was used for the predominantly screw portion of the dislocation in Fig. 3d owing to a lack of space between the dislocation and the crystal edge.

Updating the analysis previously presented in ref. [4], we can quantitatively characterize the dislocation by considering the form of their local displacement fields. For a pure edge dislocation, atomic displacements are expected only in the plane perpendicular to the dislocation line. Mathematically, the displacement field in the directions perpendicular and parallel to the extra inserted atomic plane, $u_\perp$ and $u_\parallel$ respectively, are given by:[5–8]

$$u_\perp = \frac{|\mathbf{b}|}{2\pi}\left(\theta + \frac{\sin(2\theta)}{4(1-\nu)}\right) \tag{S1}$$

and

$$u_\parallel = -\frac{|\mathbf{b}|}{2\pi}\left(\frac{\cos(2\theta)}{4(1-\nu)} + \frac{(1-2\nu)}{2(1-\nu)}\log(r)\right) \tag{S2}$$

Here, $\mathbf{b}$ is the Burgers vector that describes the dislocation and which is marked on the diagrams in Fig. 3e, and ν is the Poisson ratio of the material (taken to be 0.29; see Table S3 and associated discussion). For a pure edge dislocation, we have extracted the displacement *vs.* arc angle data from a plane containing the scattering vector, $\mathbf{Q}$, however, we do not *a priori* know the angle between $\mathbf{Q}$ and the normal of the inserted plane, which we label k. Hence, we must use a linear combination of Equations S1 & S2 to model the atomic displacement field observed, with their contributions weighted by the cosine and sine of k. *I.e.* we fit the data according to

$$u = u_\perp \times \cos(k) + u_\parallel \times \sin(k) \tag{S3}$$

where k is a refinable parameter giving the angle between $\mathbf{Q}$ and the direction normal to the extra inserted plane. Because we have used an obvious phase discontinuity to identify dislocations in the first place, in general k is small for our data. Fig. S8 outlines the coordinate system used for edge dislocation analysis. Fits of Equation S3 are shown with dashed lines in Fig. 3d.

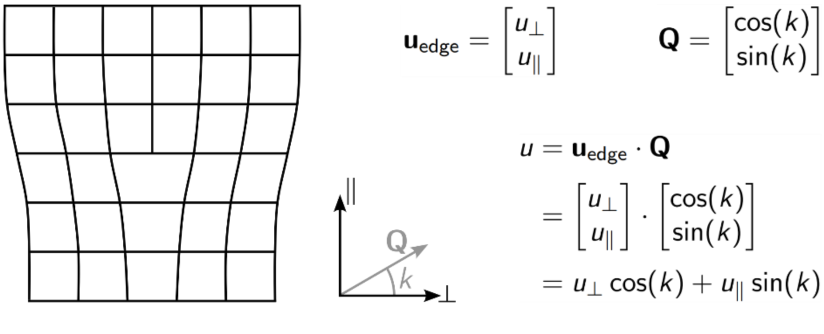

Fig. S8: Coordinate system for edge dislocation analysis.

By contrast, all the atomic displacements for a pure screw dislocation are along the direction of the dislocation line. Labelling this direction z, and this time choosing a slice through the reconstruction perpendicular to our scattering vector, the atomic displacements depend on arc angle, θ, according to[5,6,9]

$$u_{\mathrm{z}} = \frac{|\mathbf{b}|}{2\pi}\theta \qquad \text{(S4)}$$

Fits of Equation S4 are shown with dotted lines in Fig. 3d.

A pure screw dislocation will give a straight-line local displacement *vs.* arc angle plot, with that of an edge dislocation having an additional sinusoidal modulation. These facts, in combination with the phase ramps of 2π corresponding to a displacement equal to the lattice spacing of the 100 Bragg peak probed in our BCDI experiment, allows us to identify ⟨100⟩ edge and ⟨100⟩ screw dislocations by inspection. Nevertheless, the refined values of $|\mathbf{b}|$ are given in Table S2. While the agreement of $|\mathbf{b}|$ with d_{100} is not perfect, it is better than for any of the other (and smaller) d-spacings. Errors in these values may originate from compositional inhomogeneity causing local lattice parameter variation, background strain fields in addition to the dislocations', and other edge/screw character which we cannot rigorously characterise without multi-peak BCDI.

Where does the grain appear?	Fig. 3a	Fig. 3b	Fig. 3c edge	Fig. 3c screw
$|\mathbf{b}|$ /Å	6.52	5.70	7.08	7.27
difference from $\boldsymbol{d_{100}}$ /%	+3.60	−9.43	+12.50	+15.52

Table S2: Burgers vector magnitudes refined for the dislocations shown in Fig. 3 of the main text. Percent errors relative to the d_{100} lattice spacing of 6.2934Å refined from powder X-ray diffraction data presented in Fig. S1 are also given.

Finally, we require a value of Poisson's ratio, ν, for $Cs_{0.15}FA_{0.85}SnI_3$ in order to fit the data to the Equation S3. An estimate of ν is obtained for this mixed-component system using a compositionally weighted average of values for pure compositions according to a rule of mixtures as below. ν for pure compositions are tabulated in Table S3.

$$\begin{aligned}\nu(\mathrm{Cs_{0.15}FA_{0.85}SnI_3}) &= 0.15 \times \bar{\nu}(\mathrm{CsSnI_3}) + 0.85 \times \bar{\nu}(\mathrm{FASnI_3}) \\ &= 0.15 \times 0.267 + 0.85 \times\ 0.295 \\ &= 0.29075 \simeq 0.29\end{aligned}$$

Composition	$\boldsymbol{\nu}$	$\bar{\boldsymbol{\nu}}$
$FASnI_3$	0.33 [10]<br>0.26 [11]	0.295
$CsSnI_3$	0.26 [12]<br>0.28 [13]<br>0.26 [14]	0.267

Table S3: Poisson ratios for pure composition Sn-based halide perovskites from the literature and their means.

Additional experimental methods:

Powder X-ray diffraction:

X-ray diffraction data for $Cs_{0.1}FA_{0.9}Pb(I_{0.95}Br_{0.05})_3$ were performed on a Malvern Panaytical diffractometer with an angular range of 5° < 2ϑ < 70°.

X-ray diffraction measurements for $Cs_{0.15}FA_{0.85}SnI_3$ were performed in Bragg-Brentano geometry using a D8 Advance X-ray diffractometer (Bruker AXS) with a Cu-Kα source (λ=1.5418Å). Data for the experiments were collected in a locked-coupled one-dimensional mode for diffraction angles (2ϑ) between 5° and 35°, with a step size of 0.01032°.

Photoluminescence spectra:

Photoluminescence measurements were performed on a Photon Etc. IMA Vis microscope using wide-field blue (405nm) laser excitation and a 100× Olympus aberration-corrected objective lens in place. Spectral resolution is achieved since the emitted light from the sample is spectrally split through a volume Bragg grating before being collected by charged-coupled device (CCD) camera. The camera used is a 2048 × 2048 resolution Hamamatsu Orca Flash V3.0 with a wavelength range of 400–1000nm and is maintained at-10°C during measurements. The position on the sample from which light is emitted is calculated by scanning the angle of the grating relative to the emitted light. The PL emission was then summed over all pixels to give the final spectra.

Scanning electron microscopy:

Scanning electron micrographs of the $Cs_{0.1}FA_{0.9}Pb(I_{0.95}Br_{0.05})_3$ films were collected using scanning electron microscopy (SEM) (FEI Inspect-F). The surface morphology of films was characterized at 5kV and the working distance used was 10mm.

The surface of the $Cs_{0.15}FA_{0.85}SnI_3$ perovskite films was imaged using a Zeiss LEO 1550 field-emission scanning electron microscopy instrument, with a 3kV acceleration voltage. It was ensured that the voltage levels for scanning electron microscopy measurement did not notably impact the integrity of the perovskite thin films. The working distance used was 4.1mm.

JV measurements:

The current-voltage (JV) characteristics of $Cs_{0.1}FA_{0.9}Pb(I_{0.95}Br_{0.05})_3$-based devices were measured by using Keithley 2400 source-measure unit with LabVIEW 8.0 software and tested under 1 sun (100mWcm^{-2}) provided by a Newport Solar simulator apparatus.

The JV characteristics of $Cs_{0.15}FA_{0.85}SnI_3$-based devices were measured using a Sunbrick G2V LEDs solar simulator to generate illumination equivalent to 1 sun. A scan speed of 100mVs^{-1} was used for both forward and backward JV scans. The cell area was limited to 0.1182cm^2 using measurement masks.

References:

(1) Coelho, A. A. TOPAS and TOPAS-Academic: An Optimization Program Integrating Computer Algebra and Crystallographic Objects Written in C++. *J. Appl. Cryst.* **2018**, *51* (1), 210–218. https://doi.org/10.1107/S1600576718000183.

(2) Senanayak, S. P.; Dey, K.; Shivanna, R.; Li, W.; Ghosh, D.; Zhang, Y.; Roose, B.; Zelewski, S. J.; Andaji-Garmaroudi, Z.; Wood, W.; Tiwale, N.; MacManus-Driscoll, J. L.; Friend, R. H.; Stranks, S. D.; Sirringhaus, H. Charge Transport in Mixed Metal Halide Perovskite Semiconductors. *Nat. Mater.* **2023**, *22* (2), 216–224. https://doi.org/10.1038/s41563-022-01448-2.

(3) Du, T.; Richheimer, F.; Frohna, K.; Gasparini, N.; Mohan, L.; Min, G.; Xu, W.; Macdonald, T. J.; Yuan, H.; Ratnasingham, S. R.; Haque, S.; Castro, F. A.; Durrant, J. R.; Stranks, S. D.; Wood, S.; McLachlan, M. A.; Briscoe, J. Overcoming Nanoscale Inhomogeneities in Thin-Film Perovskites via Exceptional Post-Annealing Grain Growth for Enhanced Photodetection. *Nano Lett.* **2022**. https://doi.org/10.1021/acs.nanolett.1c03839.

(4) Orr, K. W. P.; Diao, J.; Lintangpradipto, M. N.; Batey, D. J.; Iqbal, A. N.; Kahmann, S.; Frohna, K.; Dubajic, M.; Zelewski, S. J.; Dearle, A. E.; Selby, T. A.; Li, P.; Doherty, T. A. S.; Hofmann, S.; Bakr, O. M.; Robinson, I. K.; Stranks, S. D. Imaging Light-Induced Migration of Dislocations in Halide Perovskites with 3D Nanoscale Strain Mapping. *Adv. Mater.* **2023**, *35* (46), 2305549. https://doi.org/10.1002/adma.202305549.

(5) Ulvestad, A.; Singer, A.; Clark, J. N.; Cho, H. M.; Kim, J. W.; Harder, R.; Maser, J.; Meng, Y. S.; Shpyrko, O. G. Topological Defect Dynamics in Operando Battery Nanoparticles. *Science* **2015**, *348* (6241), 1344–1347. https://doi.org/10.1126/science.aaa1313.

(6) P. M. Anderson. *Theory of Dislocations*, Third edition.; Cambridge University Press, ©2017: New York, NY, 2017.

(7) Ulvestad, A.; Welland, M. J.; Cha, W.; Liu, Y.; Kim, J. W.; Harder, R.; Maxey, E.; Clark, J. N.; Highland, M. J.; You, H.; Zapol, P.; Hruszkewycz, S. O.; Stephenson, G. B. Three-Dimensional Imaging of Dislocation Dynamics during the Hydriding Phase Transformation. *Nat. Mater.* **2017**, *16* (5), 565–571. https://doi.org/10.1038/nmat4842.

(8) Singer, A.; Zhang, M.; Hy, S.; Cela, D.; Fang, C.; Wynn, T. A.; Qiu, B.; Xia, Y.; Liu, Z.; Ulvestad, A.; Hua, N.; Wingert, J.; Liu, H.; Sprung, M.; Zozulya, A. V.; Maxey, E.; Harder, R.; Meng, Y. S.; Shpyrko, O. G. Nucleation of Dislocations and Their Dynamics in Layered Oxide Cathode Materials during Battery Charging. *Nat. Energy* **2018**, *3* (8), 641–647. https://doi.org/10.1038/s41560-018-0184-2.

(9) Diao, J.; Shi, X.; Assefa, T. A.; Wu, L.; Suzana, A. F.; Nunes, D. S.; Batey, D.; Cipiccia, S.; Rau, C.; Harder, R. J.; Cha, W.; Robinson, I. K. Evolution of Ferroelastic Domain Walls during Phase Transitions in Barium Titanate Nanoparticles. *Phys. Rev. Materials* **2020**, *4* (10), 106001. https://doi.org/10.1103/PhysRevMaterials.4.106001.

(10) Guo, L.; Tang, G.; Hong, J. Mechanical Properties of Formamidinium Halide Perovskites $FABX_3$ ($FA=CH(NH_2)_2$; B=Pb, Sn; X=Br, I) by First-Principles Calculations. *Chinese Phys. Lett.* **2019**, *36* (5), 056201. https://doi.org/10.1088/0256-307X/36/5/056201.

(11) Roknuzzaman, M.; Alarco, J. A.; Wang, H.; Du, A.; Tesfamichael, T.; Ostrikov, K. (K.). Ab Initio Atomistic Insights into Lead-Free Formamidinium Based Hybrid Perovskites for Photovoltaics and Optoelectronics. *Comput. Mater. Sci.* **2019**, *169*, 109118. https://doi.org/10.1016/j.commatsci.2019.109118.

(12) Shakil, M.; Akram, A.; Zeba, I.; Ahmad, R.; Gillani, S. S. A.; Gadhi, M. A. Effect of Mixed Halide Contents on Structural, Electronic, Optical and Elastic Properties of CsSnI3−xBrx for Solar Cell Applications: First-Principles Study. *Mater. Res. Express* **2020**, *7* (2), 025513. https://doi.org/10.1088/2053-1591/ab727d.

(13) Materials Data on $CsSnI_3$ by Materials Project. *LBNL Materials Project; Lawrence Berkeley National Laboratory (LBNL), Berkeley, CA (United States)* **2020**. https://doi.org/10.17188/1277770.

(14) N. Islam, M.; Podder, J.; L. Ali, M. The Effect of Metal Substitution in CsSnI<sub>3</sub perovskites with enhanced optoelectronic and photovoltaic properties. *RSC Advances* **2021**, *11* (62), 39553–39563. https://doi.org/10.1039/D1RA07609D.
